# Differential Aggregation and Phosphorylation of Alpha Synuclein in Membrane Compartments Associated With Parkinson Disease

**DOI:** 10.3389/fnins.2019.00382

**Published:** 2019-04-24

**Authors:** Ana Canerina-Amaro, Daniel Pereda, Mario Diaz, Deiene Rodriguez-Barreto, Verónica Casañas-Sánchez, Marija Heffer, Paula Garcia-Esparcia, Isidro Ferrer, Ricardo Puertas-Avendaño, Raquel Marin

**Affiliations:** ^1^Laboratory of Cellular Neurobiology, Department of Basic Medical Sciences, Section of Medicine, Faculty of Health Sciences, University of La Laguna, Santa Cruz de Tenerife, Spain; ^2^Associate Research Unit ULL-CSIC, Membrane Physiology and Biophysics in Neurodegenerative and Cancer Diseases, University of La Laguna, Santa Cruz de Tenerife, Spain; ^3^Laboratory of Membrane Physiology and Biophysics, Department of Animal Biology, Edaphology and Geology, Faculty of Sciences, University of La Laguna, Santa Cruz de Tenerife, Spain; ^4^Department of Biology, University of Osijek School of Medicine, Osijek, Croatia; ^5^Department of Pathology and Experimental Therapeutics, University of Barcelona, Barcelona, Spain; ^6^Bellvitge University Hospital, Barcelona, Spain; ^7^CIBERNED, Barcelona, Spain

**Keywords:** alpha synuclein, Parkinson disease, lipid rafts, prion protein, amyloid precursor protein, metabotropic glutamate receptor 5, NMDA receptor

## Abstract

The aggregation of α-synuclein (α-syn) is a major factor behind the onset of Parkinson’s disease (PD). Sublocalization of this protein may be relevant for the formation of multimeric α-syn oligomeric configurations, insoluble aggregates that form Lewy bodies in PD brains. Processing of this protein aggregation is regulated by associations with distinct lipid classes. For instance, instability of lipid raft (LR) microdomains, membrane regions with a particular lipid composition, is an early event in the development of PD. However, the relevance of membrane microdomains in the regulation and trafficking of the distinct α-syn configurations associated with PD remains unexplored. In this study, using 6- and 14-month-old healthy and MPTP-treated animals as a model of PD, we have investigated the putative molecular alterations of raft membrane microstructures, and their impact on α-syn dynamics and conformation. A comparison of lipid analyses of LR microstructures and non-raft (NR) fractions showed alterations in gangliosides, cholesterol, polyunsaturated fatty acids (PUFA) and phospholipids in the midbrain and cortex of aged and MPTP-treated mice. In particular, the increase of PUFA and phosphatidylserine (PS) during aging correlated with α-syn multimeric formation in NR. In these aggregates, α-syn was phosphorylated in pSer129, the most abundant post-transductional modification of α-syn promoting toxic aggregation. Interestingly, similar variations in PUFA and PS content correlating with α-syn insoluble accumulation were also detected in membrane microstructures from the human cortex of incidental Parkinson Disease (iPD) and PD, as compared to healthy controls. Furthermore, structural changes in membrane lipid microenvironments may induce rearrangements in raft-interacting proteins involved in other neuropathologies. Therefore, we also investigated the dynamic of other protein markers involved in cognition and memory impairment such as metabotropic glutamate receptor 5 (mGluR5), ionotropic NMDA receptor (NMDAR2B), prion protein (PrPc) and amyloid precursor protein (APP), whose activity depends on membrane lipid organization. We observed a decline of these protein markers in LR fractions with the progression of aging and pathology. Overall, our findings demonstrate that lipid alterations in membranous compartments promoted by brain aging and PD-like injury may have an effect on α-syn aggregation and segregation in abnormal multimeric structures.

## Introduction

Parkinson’s disease (PD) is a condition associated with aging that affects different brain areas, in particular the substantia nigra *pars compacta* (SNpc). It is also the most prevalent clinical manifestation of a family of neurodegenerative diseases characterized by an abnormal accumulation of aggregates of the protein α-synuclein (α-syn) in neurons, nerve fibers and glial cells, aptly named synucleinopathies (McCann et al., 2014). A main anatomopathological hallmark of these diseases is the accumulation of such aggregates into intracellular neuronal inclusions named Lewy bodies (LB), and dopaminergic neurons degeneration (Goedert et al., 2013). α-syn has been characterized in different configuration states from monomeric forms, fibrils and oligomers (Rochet et al., 2000; Serpell et al., 2000; Ding et al., 2002; Zarranz et al., 2004; Greenbaum et al., 2005; Lashuel et al., 2013). The predominant species of the toxic aggregates is α-syn abnormally phosphorylated on Ser-129, a pathological modification that facilitates fibril formation and insoluble aggregation (Iwatsubo, 2003; Moldovan and Moldovan, 2004; Jellinger, 2011; Samuel et al., 2016; Kumar et al., 2017). Most data agree that accumulating α-syn insoluble oligomers is a main neuropathological feature of PD and related synucleinopathies (Rockenstein et al., 2014; Burre et al., 2015; Benskey et al., 2016; Villar-Pique et al., 2016), although it is still controversial whether low size protofibrils may also cause neuronal death (Breydo et al., 2012; Lashuel et al., 2013).

Evidence has demonstrated that α-syn is present in both an unfolded cytosolic form and a membrane-bound form that assumes an α-helical conformation (Davidson et al., 1998; Kahle et al., 2000; Lee and Lee, 2002). In healthy neurons, α-syn predominantly binds to presynaptic membranes, where the protein appears to be involved in the regulation of synaptic plasticity, neurotransmitter release, vesicle transport and fusion machinery (Cabin et al., 2002; Fortin et al., 2010; Chen et al., 2013; Burre et al., 2014). Furthermore, the synaptic localization of α-syn is mediated by binding with high affinity to membrane lipid microenvironments, thereby contributing to the normal functionality of the protein (Fortin et al., 2004; Stockl et al., 2008).

Previous studies have concluded that lipid membrane interaction determines the structural modifications and functionality of α-syn (Fink, 2006). In fact, the interaction of α-syn with lipid membranes is highly dependent on both the phospholipid fatty acid composition and the ratio of the protein to phospholipid (Jo et al., 2000; Zhu and Fink, 2003). However, it is still unclear how the binding of this protein to the plasma membrane may contribute to pathological activity. For instance, physical interaction of α-syn in specific lipid microenvironments, such as LRs may determine the level of multimerization and configuration of this protein. LRs are plasma membrane microdomains with a peculiar structure and distinct lipid composition where numerous signaling proteins converge (Lingwood and Simons, 2010). These microdomains are characterized by their high content in Cho, sphingomyelin and gangliosides such as GM1. LRs are known as key points to facilitate amyloid-like seeding and toxic oligomerization associated with different neuropathologies, where it is likely that cross-seeding may be a common feature for promotion of aberrant molecular aggregates (Kakio et al., 2003; Kazlauskaite et al., 2003; Bucciantini et al., 2012; Arbor et al., 2016). Numerous publications suggest that membrane lipid composition may play a crucial role in the formation of misfolded proteins associated with proteinopathies such as PD, Alzheimer’s disease (AD), prion diseases and dementia with Lewy Bodies (DLB) (Morales et al., 2010; Busquets et al., 2015; Chu and Kordower, 2015; Espargaró et al., 2016). In particular related to synucleopathies, previous work has demonstrated that α-syn interacts with different lipid classes significantly represented in LRs, including ganglioside GM1 (Martinez et al., 2007), and PUFA, such as AA, 20:4 and DHA, 22:6 (Perrin et al., 2001; Sharon et al., 2003b). Although still poorly characterized, the integrity of LRs may be crucial for the functional integration of α-syn in membrane regions, observing a reduction of α-syn presence in presynaptic membranes following chemical disruption of LR microstructures (Fortin et al., 2004). However, it is still unknown whether membrane lipid microenvironments may have an effect on the seeding and development of α-syn oligomeric species.

Considering the preferential binding of α-syn within LR microenvironments, it is plausible that the configuration of distinct protein conformations may be regulated by specific lipid classes. Thus, preferential binding of this protein to PUFA in raft-like liposomes requires a multifactorial combination with PS and oleic acid (18:1 n-9) (Kubo et al., 2005). Interestingly, PUFA may play a role in α-syn reorganization as reflected by the increased levels of these fatty acids in α-syn knock-out mice (Sharon et al., 2003b). In support of this, association with PUFA acyl groups, such as arachidonoyl and docosahexaenoyl, appears to induce a rapid multimerization of α-syn (Perrin et al., 2001) that may promote the formation of toxic formations. In addition, other studies have demonstrated a pathological α-syn oligomerization process when binding to DHA (De Franceschi et al., 2009, 2011). Conversely, other data has evidenced that enhancement of α-syn membrane lipid binding to specific gangliosides prevents its aggregation and neurotoxicity (Burre et al., 2014). In particular, interaction of monomeric α-syn with GM1 ganglioside inhibits the protein fibrillation (Martinez et al., 2007). These results indicate that α-syn configuration in the lipid matrix may depend on the combinatorial composition of specific membrane lipid classes interacting with this protein, leading to normal or pathological formations. Adding more complexity to this scenario, the different α-syn conformations may also influence their interactions within lipid environments. In this sense, recent biophysical data have demonstrated that toxic α-syn oligomerization reduces the ability of this protein to interact with lipid membranes depending upon their chemical composition (Gallea et al., 2018). Overall, these data support the importance of neuronal membrane lipid composition and microenvironment in the regulation of α-syn conformational structure and functionality.

Our previous work has shown that LRs exhibit pathological modifications with the progression of aging that are exacerbated in age-related neuropathologies (Marin et al., 2016, 2017; Canerina-Amaro et al., 2017; Diaz et al., 2018; Marin and Diaz, 2018). In particular, aged LRs exhibit altered raft lipidomic profiles and physicochemical properties that affect protein associations and may contribute to the cell-cell propagation of toxic α-syn aggregates (Marin et al., 2017; Rodriguez et al., 2018). In this order of ideas, we have demonstrated in the human frontal cortex of both incidental and advanced PD subjects, the presence of lipid changes in LRs that correlate with the progression of the disease (Fabelo et al., 2011; Diaz et al., 2018). Noticeably, even in asymptomatic incidental PD (iPD) brains, there was a detectable reduction of AA and DHA, in parallel with an increase of saturated fatty acids (SFA, 16:0 and 18:0). These alterations induce a loss of stability in LRs that correlates with variations of the peroxidability and unsaturation indices among other factors and an increase of viscosity (membrane-ordered) microstructure (Fabelo et al., 2012). The perturbations in the physicochemical properties of LRs enhance aberrant signaling protein rearrangements, amyloidogenesis, amyloid aggregation and toxic cell signaling in aged-associated neurodegenerative diseases (Rushworth and Hooper, 2010; Schengrund, 2010; Diaz et al., 2015; Marin et al., 2016). These findings reflect the relevance of lipid microstructures in the pathological progression of PD (Kubo et al., 2015).

Another consequence of altered structure of LRs during neuropathology is that they may modify the interactions of raft-integrated multiprotein associations. A big body of data indicates that aberrant oligomerization of key self-aggregating protein markers in different proteionopathies, such as amyloid beta (Ab), the prion protein (PrPc) and α-syn, may form multicomplexes with other membrane-related proteins, thus contributing to neuropathological features. In this sense, α-syn has been found associated with PrPc, a phenomenon involved in synaptic dysfunction (Ferreira et al., 2017). This interaction mediates the activation of NMDAR2B through mGluR5 triggering early synaptic and cognitive deficits in mice. These multimeric signaling platforms may also be modulated by the biochemical interrelations with membrane-integrated molecular compounds (Goedert, 2015; Ugalde et al., 2016). Thus, PrPc tightly binds to ganglioside GM1 which is preferentially found in LR membrane microstructures (Sanghera et al., 2011). For instance, it has been postulated that GM1 may act as a scaffolding molecule that provides stabilization of this protein in the lipid physicochemical environment (Botto et al., 2014).

Taking into account previous data, it is relevant to investigate the potential correlation of alterations in the membrane binding of α-syn with membrane lipid structure aberrations generated during PD progression that still remain unexplored. Here, using the MPTP mouse model of PD, we have analyzed the consequences of PD-like pathology and aging on either α-syn distribution or configuration in membrane biochemical fractionation of the midbrain, cortex and cerebellum. We selected cortical regions based upon our previous observations in human brains where profound changes in lipid profiles were detected (Marin et al., 2017; Diaz et al., 2018). Both the cortex and the midbrain have been characterized as the main brain regions affected by PD pathology. Indeed, cognitive impairment is one of the early clinical features of PD, occurring before motor symptoms. For comparison, the cerebellum was also included in this study. Furthermore, LRs isolated from human frontal cortex of asymptomatic incidental PD (iPD) and PD brains were also investigated to further validate the impact of the findings in the animal model.

The results demonstrate that both aging and neurotoxic treatment modify lipid profiles of membrane microdomains, in correlation with an increase in multimeric α-syn aggregation and phosphorylation. In addition, membrane lipid impairment may also affect the partitioning of other protein markers involved in the neuropathology, such as PrPc, mGluR5, NMDAR2B, and APP.

## Materials and Methods

### Materials

MPTP-HCl, Cholera toxin B subunit-horseradish peroxidase (HRP) and SIGMAFAST DAB with Metal Enhancer were purchased from BioSigma (Tenerife, Spain). The rabbit polyclonal antibody against TH, and mouse monoclonal antibody 5G4 against aggregated α-syn were purchased from Millipore (Madrid, Spain). The Dynabeads Coupling Kit was from Thermo Scientific (Madrid, Spain). The mouse monoclonal antibodies against synuclein 4D6 clone, anti-amyloid precursor protein (APP), the rabbit polyclonal antibodies against, respectively, pSer129 α-syn, NMDAR2B, Flotillin-1, and the rabbit monoclonal antibody against alpha synuclein [EPR20535], and anti-mGluR5 were from Abcam (Cambridge, United Kingdom). The mouse monoclonal anti-amyloid beta peptide antibody and the monoclonal anti-PrPc were purchased from Santa Cruz Biotechnologies (Texas, EEUU). The mouse monoclonal antibodies against GD1a-1, GD1b-1 and GT1b-2b were from Ronald L. Schnaar laboratory (Schnaar et al., 2002). PreCast Mini-Protean SDS-PAGE gels, immobilized pH3-10 gradient strips for two-dimensional gel electrophoresis, Trans-Blot^®^ Turbo^TM^ Mini PVDF Transfer Packs, Blotting Grade Blocker, ReadyPrep 2-D Cleanup Kit and Clarity^TM^ Western ECL Substrate were from Bio-Rad Laboratories (Madrid, Spain). The phosphoBLOCKER blocking reagent and the phospho antibody stripping solution were from Cell Biolabs, Inc (Madrid, Spain). The Complete protease inhibitor cocktail was purchased from Roche Diagnostics (Barcelona, Spain).

### Animal Handling and Treatment

Six-months- and fourteen-months-old female C57BL/6J mice weighing 25-30 g were housed in enriched cages with food and water *ad libitum*, and maintained under a 12-h light/dark cycle in the Animal Facilities at La Laguna University. All procedures were performed in accordance with the European Community Directive (86/609/UE) and Universidad de La Laguna Ethics Committee guidelines for the care of laboratory animals.

Four experimental groups were established. Six-months and 14-months-old mice received daily injections of 4 mg MPTP/kg/day for 20 days (respectively, **M6** and **M14**). Age-matched controls of these two groups were treated with saline (respectively, **W6** and **W14**). Mice were deeply anesthetized and sacrificed 43 days after finishing the treatment. MPTP-treatment method has been shown to induce a progressive dopaminergic cell loss (Meredith and Rademacher, 2011). For LRs isolations tissue was immediately dissected and frozen. Four animals per experimental group were used. For immunohistochemically analysis, animals were perfused with saline and 4% r-formaldehyde. Tissues were post-fixed in 4% r-formaldehyde for 24 h and processed to be included in paraffin block processing of neural tissues. Three animals per experimental group were used.

### Human Brain Samples

Human samples were acquired from the Institute of Neuropathology Brain Bank (Bellvitge University Hospital) following legal and ethical guidelines for Biomedical Research involving human subjects (Declaration of Helsinki) and approval of the local Ethics Committee. Autopsies were conducted 3–12 h post-mortem. Brains were bisected into hemispheres with one hemisphere immediately store at -80°C until used for molecular studies. LR isolation was processed in the Institute of Neuropathology Brain Bank following standard protocols (Mukherjee et al., 2003) with slight modifications previously described by our group (Martin et al., 2010; Fabelo et al., 2011). The frontal cortex of five cases with PD (average age 72.8 years), five cases with incidental PD (average 71.2 years), and six age-matched controls with no neurological symptoms and signs (age 75.2 years) were analyzed. Subjects with iPD did not report neurological anomalies, although PD-related neuropathological markers were found in the post-mortem study.

### Immunohistochemistry

Mice whole brain samples were coronally cut into 10 μm sections with a paraffin microtome, and mounted on glass slides pretreated with (3-Aminopropyl)-triethoxysilane.

Slides were deparaffinized by immersion in xylene (2x 15 min), and rehydrated using graded ethanol (2x 100% for 5 min; 95% for 5 min; 90% for 5 min; 80% for 5 min; 70% for 5 min), followed by washes with distilled water (2x 5 min) and phosphate-buffered saline (PBS, 2x 5 min). For antigen retrieval slides were boiled for 15 min in 20 mM citrate buffer (pH 6). After three washes with tris-buffered-saline (TBS), slides were incubated overnight at room temperature (RT) with primary antibody for TH diluted 1:500 in TBS. Slides were washed three times in TBS, and then incubated 2 h at RT with the specific secondary-HRP antibody diluted 1:200 in TBS. After washing three times with TBS, Signal was developed using SIGMAFAST DAB (3,3′-Diaminobenzidine tetrahydrochloride) with metal enhancer (Sigma Aldrich). After washing in distilled water, slides were dehydrated in graded ethanol dilutions, and xylene and permanently mounted with Eukitt (O. Kindler, Freiburg, Germany).

### Quantification of TH+ Cells in the SNpc

Sections comprising the substantia nigra *pars compacta* were selected for immunohistochemistry with TH. Those sections that produced positive staining were photographed, and positive cells were counted with ImageJ software. For each animal evaluated, total number of positive cells was calculated by adding up the number of cells in each section.

### Isolation of Lipid Rafts

Lipid raft isolation was performed according to previous protocols (Mukherjee et al., 2003) with slight modifications (Martin et al., 2010). Cortex (Co), midbrain (Md), and cerebellum (Cb) were homogenized in buffer A (50 mM Tris-HCl, pH 8.0, 10 mM MgCl_2_, 150 mM NaCl), containing 1% Triton X-100 and 5% glycerol, and supplemented with 20 mM NaF, 1 mM Na_3_VO_4_, 5 mM β-mercaptoethanol, 1 mM PMSF, and complete protease inhibitor. All the steps were performed on ice. Brain homogenates were centrifuged at 500 *g* for 5 min at 4°C, and the supernatant was collected and mixed in an orbital rotor for 1 h at 4°C. Sucrose solutions of 80, 36, and 15% were prepared in buffer A. About 800 mL of each homogenate were added to an equal volume of 80% sucrose solution, and overlaid with 7.5 mL of a 36% sucrose solution and 2.7 mL of a 15% sucrose solution, in ultracentrifuge tubes (Ultraclear, Beckman, Izasa, Tenerife, Spain). Sucrose gradients were centrifuged at 150,000 *g* for 18 h at 4°C in a Beckman SW41Ti rotor. Fractions (F1–F6) of 2 mL were collected from the top to the bottom and the final pellet were collected, and resuspended in 200 mL of buffer A, and stored at -80°C. Fractions F1 and F2 corresponded to LR microdomains, whilst pellet (F6) comprised the non-raft fraction. Protein contents in each fraction were determined by the bicinchoninic acid (BCA) method and western blot assays were conducted in order to characterize the different fractions.

### Slot-Blot Analysis

For gangliosides and glutamatergic receptors detection, corresponding volumes for 200 ng of total protein of each gradient fraction were spotted onto a nitrocellulose membrane sealed on a Slot-blot set-up (Bio-Rad). Membranes were blocked with TBS containing 3.5% (w/v) of bovine serum albumin (BSA) for 1 h at RT. For each ganglioside and glutamatergic receptor analyzed one membrane was spotted. The immunodetection of GD1a, GD1b, and GT1b gangliosides was performed by incubation with specific mouse monoclonal antibodies overnight at 4°C, followed by incubation with the corresponding secondary-HRP antibody. Rabbit monoclonal anti-mGluR5 antibody and rabbit polyclonal Anti-NMDAR2B antibody were used for the immunodetection of corresponding receptors. For the GM1 ganglioside detection, membranes were incubated with the cholera toxin B subunit-HRP (1/20000, Sigma Aldrich) for 45 min at RT. In all cases, signal was developed with Clarity^TM^ Western ECL Substrate. Detection was performed with Chemie-Doc MP Imaging System (Bio-Rad), and its optical density analyzed using Image Lab software.

### Lipid Analysis

Lipid analyses were performed following previously described protocol (Fabelo et al., 2011). Briefly, total lipids from LR and NR fractions were extracted with chloroform/methanol (2:1 v/v) containing 0.01% of butylated hydroxytoluene (BHT) as an antioxidant. Only gray matter was used. The organic solvent was evaporated under a stream of nitrogen, and the lipid content was determined gravimetrically. Lipid classes were separated by one-dimensional double development high performance thin layer chromatography (HPTLC). Two different solvents were used depending on the lipid classes to be extracted: methyl acetate/isopropanol/chloroform/methanol/KCl 0.25% (5:5:5:2:1.8 v/v) for the polar lipid classes, and hexane/diethyl ether/acetic acid (22.5:2.5:0.25 v/v) for the neutral lipid classes. For quantification, plates were processed for charring with 3% (w/v) aqueous cupric acetate containing 8% (v/v) phosphoric acid. Lipid classes were quantified by scanning densitometry, using a Shimadzu CS-9001PC dual wavelength spot scanner. Equal amounts of total lipids were used in all analyses.

Fatty acids composition was determined from total lipids in the fractions upon acid-catalyzed transmethylation for 16 h at 50°C, using 1 ml of toluene and 2 mL of 1% sulfuric acid (v/v) in methanol. The resultant fatty acid methyl esters (FAME) and dimethyl acetals (DMA) were purified in thin layer chromatography (TLC), and quantified using a TRACE GC Ultra (Thermo Fisher Scientific, Waltham, MA, United States) gas chromatograph equipped with a flame ionization detector. Individual FAME and DMA were identified by reference to proper standards.

### SDS-PAGE and Two-Dimensional Gel Electrophoresis

Lipid raft fractions containing approximately 30 mg of protein were cleaned with readyPrep 2-D cleanup Kit (Bio-Rad) and resuspended in DeStreak rehydration solution (GE Healthcare) in a total volume of 125 μL. Samples were applied to immobilized pH gradient 7 cm-length strips pH 3-10, and processed for isoelectrofocusing (IEF) up to 20,000 V/h. Then, IEF strips were reduced in equilibration buffer containing 2% (w/v) dithiothreitol (DTT) for 15 min at room temperature, followed by alkylation in equilibration buffer containing 2.5% (w/v) iodoacetamide for 15 min at room temperature. Strips were loaded onto 12.5% SDS-PAGE for second dimension resolution. In other set of experiments, protein extracts were resuspended in loading buffer for SDS-PAGE electrophoresis.

### Immunoblotting

Proteins resolved by SDS-PAGE were transferred to polyvinyl (PVDF) membranes using the Trans-Blot Turbo rapid western blotting transfer system (Bio-Rad, Madrid, Spain). PVDF membranes were blocked with 5% BLOTTO (non-fat dried milk in TBS plus 0.1 Tween-20). Then, membranes were incubated overnight at 4°C with the different primary antibodies (mouse monoclonal anti-aggregated α-synuclein (5G4 clone), anti-α-synuclein (4D6 clone), anti-PrPc, anti-Aβ peptide, and anti-APP antibodies; the rabbit polyclonal anti-pSer129 α-syn and anti-Flotillin-1 antibodies; and the rabbit monoclonal (anti-α-synuclein [EPR20535] antibody). All antibodies were diluted 1:1,000 in BLOTTO, except antibodies purchased from Santa Cruz biotech that were diluted 1:200 according to manufacturer’s indications. Membranes were washed three times for 5 min in TBS with 0.1% Tween-20. Proteins were detected using the corresponding peroxidase-conjugated anti-mouse or anti-rabbit secondary antibody diluted 1:5,000 for 1h at room temperature. Specific bands were developed with Clarity^TM^ Western ECL Substrate and processed using Chemie-Doc MP Imaging System (Bio-Rad). The optical density was analyzed using Image Lab software.

### Statistical Analyses

Numerical data were represented either as mean ± SEM or normalized to young, healthy control (W6), based on clarity. Statistical significance and statistical analysis performed is detailed in every figure, as calculated in GraphPad Prism 8 statistical software. Briefly, One-way ANOVA with Newman–Keuls multiple comparison test was used when comparing experimental cohorts, and Two-way ANOVA with Fisher’s LSD when comparing the effect of aging and treatment in different brain regions. Group size also varies for different experiments, and it has been indicated in figure legends.

## Results

### Loss of TH Within SNpc Induced by MPTP Lesion

**FIGURE 1:**
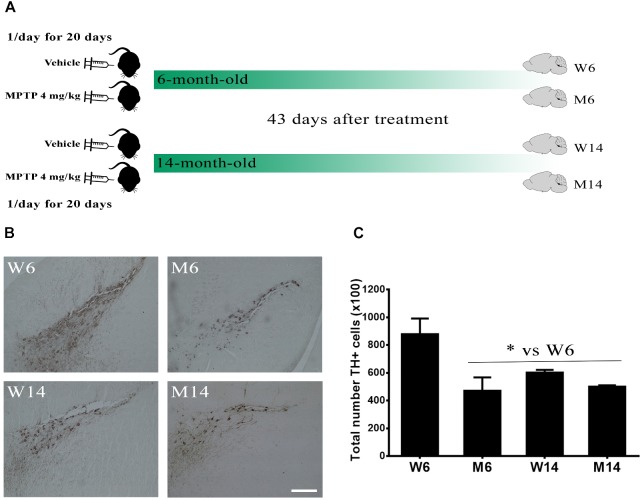
Characterization of the MPTP neurotoxicity in the animal model. **(A)** Schematic timeline of the experiments. 6-month- and 14-month-old littermate animals were injected with either saline vehicle or 4 mg/kg MPTP once a day for 20 days and their brains extracted 43 days after the end of the treatment, thus creating four experimental cohorts. **(B)** Immunostaining against TH in the substantia nigra *pars compacta* region of wild type (W) and MPTP-treated (M) mice for two different age cohorts, 6- and 14-month-old (W6, M6, W14, and M14). **(C)** Quantification of TH-immunostaining in the different experimental groups (^∗^*p* < 0.05, One-way ANOVA with Newman–Keuls multiple comparison test vs. W6 (*N* = 3). White bar = 100 μm.

The substantia nigra *pars compacta* (SNpc) is a brain area highly affected in PD pathology, with a significant reduction of dopaminergic neurons. Thereby, we first evaluated the effects of MPTP hydrochloride treatment (4 mg/kg once a day for 20 days) in aged (14 months old) mice as compared to young (6 months old) ones. We established four experimental mice groups: **W6,** control 6-months old mice; **M6,** MPTP-treated 6-months old mice; **W14,** control 14-months old mice; and **M14,** MPTP-treated 14-months old mice (Figure 1A). The total TH-IR was determined for each mouse in the different brain slices stained with anti-TH antibody (>20 slices per animal). Then, average values for each group were calculated (*n* = 3/group). A significant reduction of TH-IR (>20%) was observed in MPTP-treated mice since 6-months age, also detecting similar reduction values of 14-months old mice in both control and MPTP-treated groups (Figure 1B,C).

These results indicate that MPTP treatment and, to a lesser extent, aging, induce a significant reduction of dopaminergic activity in SNpc, thus emulating significant injury occurring during PD pathology.

### Alterations in Gangliosides and PUFA Content in Lipid Rafts by MPTP Treatment

Gangliosides are highly abundant in LRs as part of their intrinsic structure. Numerous raft-integrated proteins are known to be tightly associated with these lipids. To investigate the potential alterations of the brain major ganglioside classes, GM1, GD1a, GD1b, and GT1b following MPTP neurotoxicity, LR and non-raft (NR) fractions of the different age-and MPTP-treated experimental groups (W6, M6, W14, and M14) were explored. Membrane fractions were extracted from Md, Co, and cCb. Md and Co are main brain regions affected in PD. Cb was used as a control, as the membrane lipid profiles of this part of the CNS have been shown to remain invariable (Fabelo et al., 2011).

**FIGURE 2:**
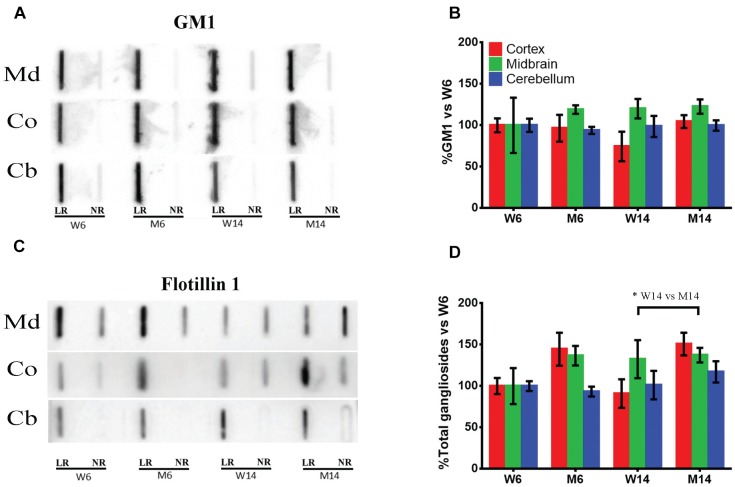
**(A)** Slot-blot analysis of ganglioside GM1 in lipid raft (LR) and non-raft (NR) fractions. Three different CNS functional areas were studied in the four experimental cohorts: midbrain (Md), cortex (Co) and cerebellum (Cb), with normalization vs. W6 values used as a control. **(B)** Quantification of ganglioside GM1 levels in LR isolated from the same three CNS areas normalized vs. W6. **(C)** Slot-blot analysis of scaffolding protein flotillin1 in the same regions. **(D)** Quantification of total ganglioside (GM1 + GD1a + GD1b + GT1b) levels in LR normalized vs. W6. ^∗^*p* < 0.05, Two-way ANOVA with Fisher’s LSD (*N* = 4).

In these membrane samples, we analyzed by slot blot the potential changes in ganglioside content using specific antibodies specifically directed against the main brain ganglioside species (GM1, GD1a, GD1b, and GT1b). The sum of these four gangliosides was also analyzed in order to have an approximate idea of total gangliosides. Cholera toxin coupled to HRP was used to visualize GM1 amount, as this toxin is known to bind specifically to GM1 on the membrane surface (Figure 2A; see Supplementary Figure S1 for the results of all four animals used for quantification). Moreover, an antibody directed against LR scaffolding protein flotillin-1 was used as a protein marker of rafts for value normalization (Figure 2B). Additionally, we normalized immunosignals vs. young healthy controls (W6). The slot blots showed that the ganglioside GM1 was exclusively localized in LR, with no significant differences found between the different nervous regions or experimental cohorts relative to flotillin 1 (Figure 2C). In contrast, total ganglioside distribution in LR was significantly altered following MPTP-related neurotoxicity (Figure 2D). In particular in Co (red), we observed an increase of the total ganglioside amount following MPTP treatment. Although not statistically significant, we also observed a marked upward trend in Md associated with age and neurotoxicity (Figure 2D, green). These data indicate that the neurotoxic treatment affects the ganglioside partitioning in micromembrane structures.

### Nerve Tissue Lipid Classes and Fatty Acids in Membrane Distinct Fractions

To further explore the potential changes occurring in the lipid matrix following mice injury and aging, we next analyzed the lipid composition of the different nerve fractions in aged MPTP mice. The complete analysis of the different lipid classes and fatty acids from the three nerve regions are detailed in Supplementary Figures S2, S3. Midbrain lipid matrix, in comparison with Co and Cb, was highly affected by MTP-related neurotoxicity. Co and Cb also showed a similar trend decrease in their relative lipid amount.

**FIGURE 3:**
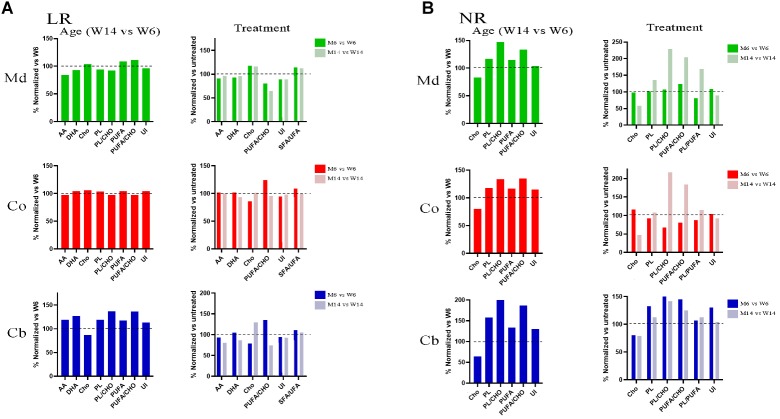
**(A)** Lipid profiles of midbrain (Md) cortex (Co) and cerebellum (Cb) across the experimental cohorts in lipid raft (LR) fractions, following aging (M6 vs. W6, left) and MPTP treatment (M6 vs. W6 and M14 vs. W14, right). **(B)** Lipid profiles of midbrain (Md) cortex (Co) and cerebellum (Cb) across the experimental cohorts in non-raft (NR) fractions in aged (M6 vs. W6, left) and treated (M6 vs. W6 and M14 vs. W14, right) cohorts (*N* = 4).

A careful analysis of the extensive raw data revealed relevant changes in specific lipid classes and fatty acids of LRs during aging and MPTP phenotype as compared with young healthy mice, summarized as follows (Figure 3A): sulfated galactosylceramides (sulfatides) were exclusively present in LR fractions. This is an indicative of the purity of LR extraction, as previously described (Moyano et al., 2014). Remarkably, we observed an increase in Cho during aging and in response to pharmacological insult. Conversely, the levels of the most abundant PUFA in nerve cells, DHA and AA, were diminished. The total phospholipid content was also lower in aged and treated animals. Moreover, the proportion of PUFA versus Cho content significantly varied, detecting a detriment in aged and MPTP animals. This was in parallel with an increase in the ratio SFA/UFA. As a consequence of the fluctuations in SFA and PUFA content, unsaturation index (UI) was reduced in aged and MPTP animals as compared to young controls. These findings are in line with previous analyses in LR from human cortical areas in both PD and LBD synucleopathies (Fabelo et al., 2012; Marin et al., 2017).

In the aim of establishing a comparative analysis of the lipid profiles between the membrane biochemical partitioning, we next performed a comparative analyses of NR lipid profiles (Figure 3B). Noticeably, we found a Cho downward and PUFA upward trend following aging and MPTP treatment that inversely correlated the one found in LR in all three regions. Indeed, Cho was depleted whereas PUFA showed higher content in NR fractions. Furthermore, there were higher levels of total phospholipids (PL), PS in particular, with aging and after treatment in aged mice. Interestingly, MPTP-induced injury generated a detectable increase in PUFA/Cho, PL/PUFA and PL/Cho ratios of aged MPTP animals in all three regions as well, particularly in aged mice.

**FIGURE 4:**
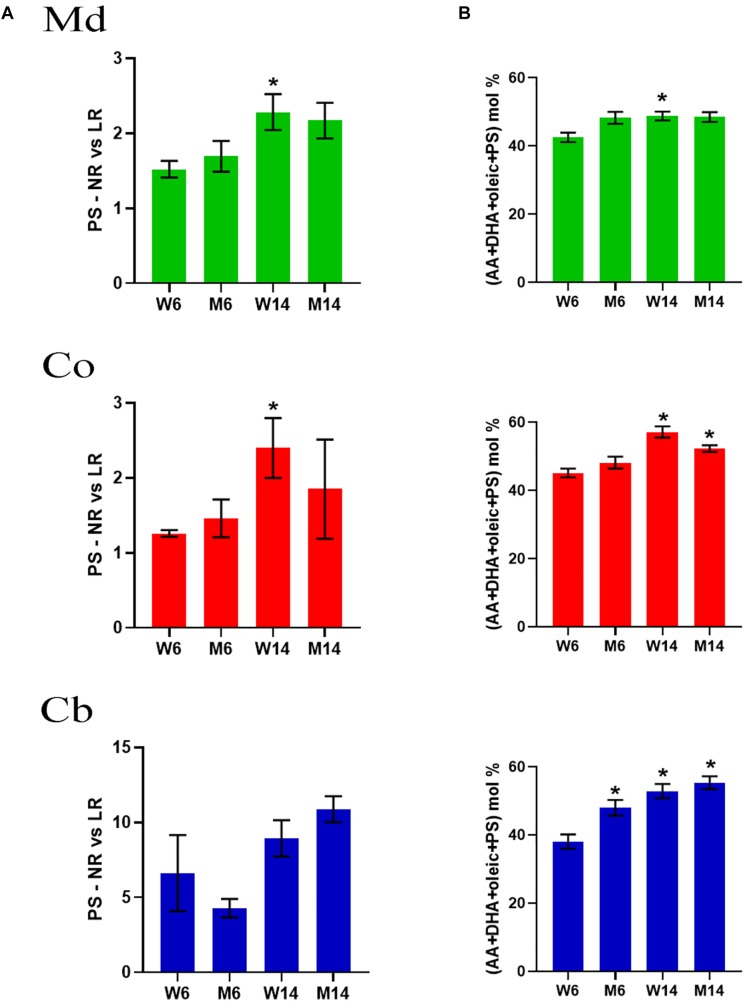
**(A)** PS levels in midbrain (Md) cortex (Co) and cerebellum (Cb) across the experimental cohorts in non-raft (NR) vs. its lipid raft (LR) fractions (^∗^*p* < 0.05, One-way ANOVA with Newman–Keuls multiple comparison test vs. W6). **(B)** Cumulative mole percentage of arachidonic acid (20:4n-6) and docosahexanoic acid (22.6n-3) (AA and DHA, respectively), oleic acid (18:1n-9) and phosphatidylserine (PS) in NR fractions from midbrain (Md) cortex (Co) and cerebellum (Cb). ^∗^*p* < 0.05, One-way ANOVA with Newman–Keuls multiple comparison test vs. W6 (*N* = 4). No significant differences were found between treated and saline littermates.

Previous work has demonstrated that lipid interaction with α-syn requires a PS combination with PUFA (AA and DHA) and oleic acid (18:1n-9) (Kubo et al., 2005), a phenomenon that triggers the rapid multimerization of α-syn (Perrin et al., 2001; Sharon et al., 2003a; Lucke et al., 2006). To explore whether mice aging or PD-like treatment may induce reassemblies of these lipid molecules, we analyzed the relative proportions of PS, DHA and oleic acid in nerve membrane microdomains. Interestingly, there was an upward trend in PS changes in Md and Co nerve regions induced by aging (Figure 4A). For instance, PS showed a marked increase in NR in all three nerve regions, as manifested by the increased PS NR/LR ratio.

Moreover, the net percentage of PS, DHA and oleic acid revealed a significant rise in the content of these lipid classes that accounted for over the ∼60% of total lipid impairment (Figure 4B). Level changes were exclusive of PS, not observing a combinatory significant trend with any other phospholipids (data not shown). Intriguingly, a simple mathematical analysis of the statistical values showed that, despite an increasing average, the standard deviation of these data decreased (data not shown).

We further explored the potential bivariate relationships between PUFA (DHA+AA) and PS in these LRs (SQR(DHA+AA)^∗^PS. The results indicated that, in particular in Md and Co of aged animals, MPTP-induced neurotoxicity induced a linear dissociation between PS and PUFA as compared to young control mice (Supplementary Figure S4).

### Increased Membrane Accumulation of α-syn Multimeric Formations With MPTP Neurotoxicity and Aging

Previous data has shown that α-syn is tightly bound to lipid species highly represented in LR microstructures, including gangliosides and PUFA (Kubo et al., 2005; Martinez et al., 2007). These distinct interactions with particular lipid classes may regulate α-syn configuration at the neuronal membrane (Burre et al., 2015) However, whether PD-related raft lipid alterations may enhance abnormal α-syn aggregation remains uncharacterized.

In order to explore whether MPTP PD-like toxicity may promote changes in α-syn configuration in correlation with lipid changes in membrane microstructures, we analyzed in both LR and NR fractions of the distinct experimental mice groups (W6, M6, W14, M14) the presence of α-syn monomeric and multimeric forms. We performed Western Blot (WB) analyses in protein extracts from Md, Co and Cb using specific antibodies that recognize distinct α-syn configurations (see Materials and Methods). We opted for normalizing WB immunosignals vs. young healthy controls’ (W6) values to better reflect changes in distribution and configuration of α-syn in response to age and treatment. Therefore, the statistical significance refers to the magnitude of the relative change of α-syn amount vs. control.

**FIGURE 5:**
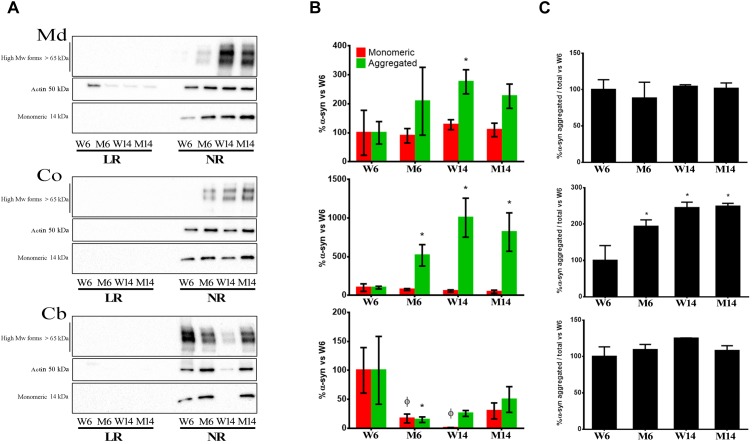
**(A)** Immunoblotting distribution of monomeric, oligomeric (anti- alpha synuclein [EPR20535] antibody, from Abcam) and aggregated α-syn (5G4 antibody, from Millipore) forms in midbrain (Md) cortex (Co) and cerebellum (Cb) across the experimental cohorts in lipid raft (LR) and non-raft (NR) fractions. **(B)** Quantification of monomeric and aggregated α-syn forms in non-raft fractions (since this protein was absent or only slightly represented in lipid rafts) from the immunoblotting assay from the same three CNS functional areas normalized versus W6. Notice that the graph shows WB immunosignals normalized vs. young healthy controls’ (W6) values to better reflect changes in distribution and configuration of α-syn in response to age and treatment. Therefore, the statistical significance refers to the magnitude of the relative change of α-syn amount vs. control. Actin immunoblotting bands are included as loading controls). ^∗^*p* < 0.05, unpaired *t*-test with Welch’s correction; ^?^*p* < 0.05, Two-way ANOVA with Fisher’s LSD. **(C)** Percentage of aggregated α-syn vs. total α-syn. ^∗^*p* < 0.05, One-way ANOVA with Newman–Keuls multiple comparison test vs. W6 (*N* = 4).

The results showed that monomeric α-syn was visualized as the 14 kDa band (Mor et al., 2016). This form was abundant in NR in all three CNS functional areas studied (Figure 5A,B). Moreover, we found an increase of multimeric α-syn forms with both aging and treatment, in particular in Co (Figure 5C, middle). NR fractions of Md also exhibited a similar increase in the presence of α-syn multimers (Figure 5A,B). In contrast, Cb levels of the protein significantly decreased in NR with age and treatment (Figure 5A,B).

### pSer129 α-syn Phosphorylation Correlates With α-syn Oligomerization in NRs

Phosphorylation of α-syn at Ser129 has been associated with α-syn aggregation and Lewy bodies generation (Fujiwara et al., 2002; Anderson et al., 2006; Xu et al., 2015). Given the aforementioned abundance of multimeric α-syn forms in Co and Md regions of MPTP and aged mice, we next analyzed α-syn phosphorylation at Ser129 in NRs of mice cohorts.

**FIGURE 6:**
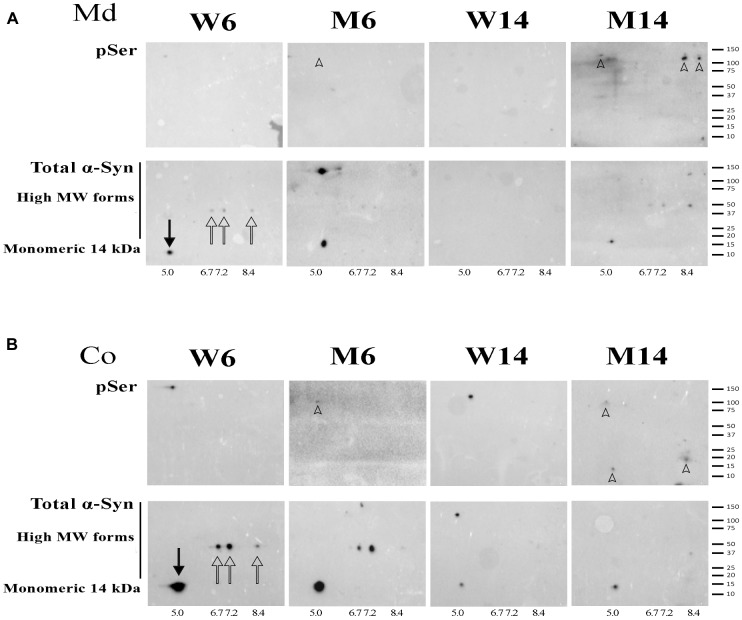
**(A)** 2-D immunoblotting of non-raft (NR) fractions showing the pattern of isoforms of α-syn in the midbrain (Md) area of the experimental cohorts. **(B)** 2-D immunoblotting of NR fractions showing the pattern of isoforms of α-syn in the cortical (Co) area of the experimental cohorts. Protein extracts of the distinct neuronal membrane fractions were processed for 2-D analyses, immunoblotted with an antibody against phospho-Ser129 α-syn (indicated as pSer), and reblotted with an antibody that recognized monomeric and high Mw α-syn forms. Isoelectric points were estimated for the native (solid arrow) and three other recurring forms (hollow arrows). High Mw, phospho-Ser α-syn isoforms were found in the M14 cohort and are highlighted (arrowheads). A summary of the molecular weight marker run in the gels is included for further reference (*N* = 4).

NR fractions of the two brain areas of 6-months and 14-months old MPTP-treated mice (M6 and M14, respectively) and age-matched controls (W6 and W14, respectively) were used to double run on two-dimensional electrophoresis using pH 3-10 non-linear gradient strips. 2-D membranes were immunoblotted with anti-pSer129 α-syn antibody, and reblotted with anti α-syn (4D6 clone) antibody. α-syn was resolved in 4 predominant isoforms with calculated isoelectric points (p*I*) of 5.0, 6.7, 7.2, and 8.4 in W6 NR pellets (Figure 6A,B, arrows). To the best of our knowledge, this is the first description of α-syn isoforms pattern in neuronal membranes. The 5.0 p*I* form was the predominant α-syn isoform detected in NR pellets corresponding to monomeric α-syn (solid arrow). This isoform was attenuated as a result of MPTP and aging, in parallel with the increased accumulation of higher Mw α-syn isoforms (arrowheads).

NR extracts were further processed for 2-D immunoblotting with anti-pSer129 α-syn antibody in order to evaluate the potential increase in α-syn phosphorylation related to protein oligomerization. We found the accumulation α-syn phosphorylated residues preferentially in high Mw forms of α-syn, especially in old mice treated with MPTP (arrowheads). Moreover, in particular in Md areas, the phosphorylated residues overlapped with α-syn multimeric forms (Figure 6A, arrowheads). Interestingly, no pSer129 residues were detected in young healthy mice (W6).

These data suggest that pSer129 α-syn phosphorylation accumulates in α-syn oligomeric conformations of NR fractions. Indeed, the activation of α-syn phosphorylation appears to be related to brain aging and is exacerbated with MPTP-related neuropathology.

### NMDAR2B and mGluR5 Membrane Redistribution in Response to Aging and Treatment

**FIGURE 7:**
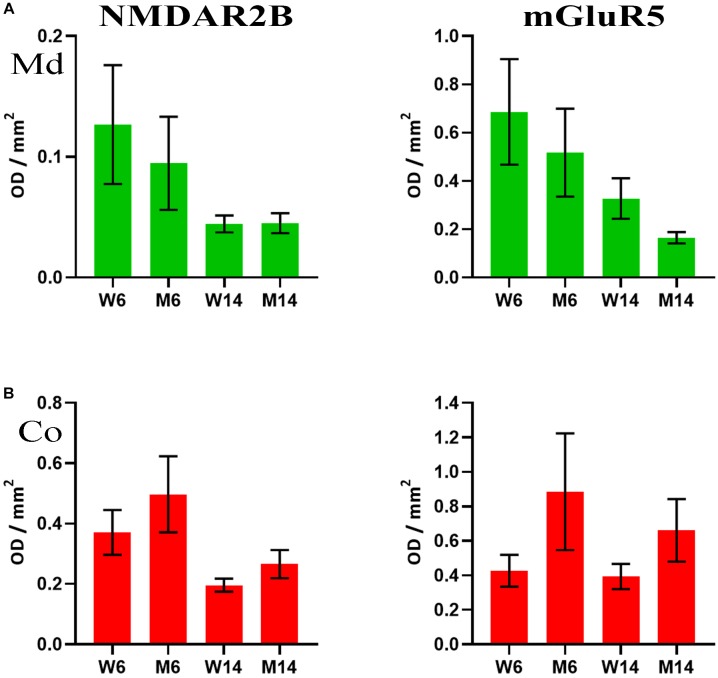
**(A)** Quantification of NMDAR2B (left) and mGluR5 (right) receptors as analyzed by slot-blot of lipid raft (LR) fractions of midbrain (Md) in our four experimental cohorts. Values were normalized vs. flotillin-1. **(B)** Quantification of NMDAR2B (left) and mGluR5 (right) receptors as analyzed by slot-blot of lipid raft (LR) fractions of cortex (Co) in our four experimental cohorts. Values were normalized vs. flotillin-1. One-way ANOVA with Newman-Keuls multiple comparison test vs. W6 (*N* = 4). No significant differences were found between treated and saline littermates, or between young and aged animals.

The accumulation of α-syn oligomers was recently shown to cause synaptic impairment through a complex mechanism involving the ionotropic NMDAR2B and the metabotropic mGluR5 receptors, as well as a direct interaction with the prion protein (Ferreira et al., 2017). This mechanism seems to be key for cognitive decline associated to synucleinopathies. Given our previous findings regarding α-synuclein, we next investigated the potential changes in NMDAR2B and mGluR5 in our experimental cohorts. We analyzed by slot-blot the presence of both receptors and optical density was normalized against flotillin-1. Although the data did not reach a standard level of significance (*p* = 0.08), both receptors showed lower levels in LRs of, both Co and Md regions following MPTP toxicity (Figure 7A,B). Slot-blotting was used for these analyses, due to the poor protein material generally extracted from LR fractions. It is worth mentioning that these findings were specific to these two, and no other, glutamatergic receptors. For instance, immunoblotting signals for the AMPA-subtype glutamate receptor 1, another ionotropic glutamatergic receptor, and mGluR1, a metabotropic receptor closely related to mGluR5, remained invariable in both fractions (data not shown).

### Membrane Redistribution of PrPc Associated With Aging and MPTP Treatment

**FIGURE 8:**
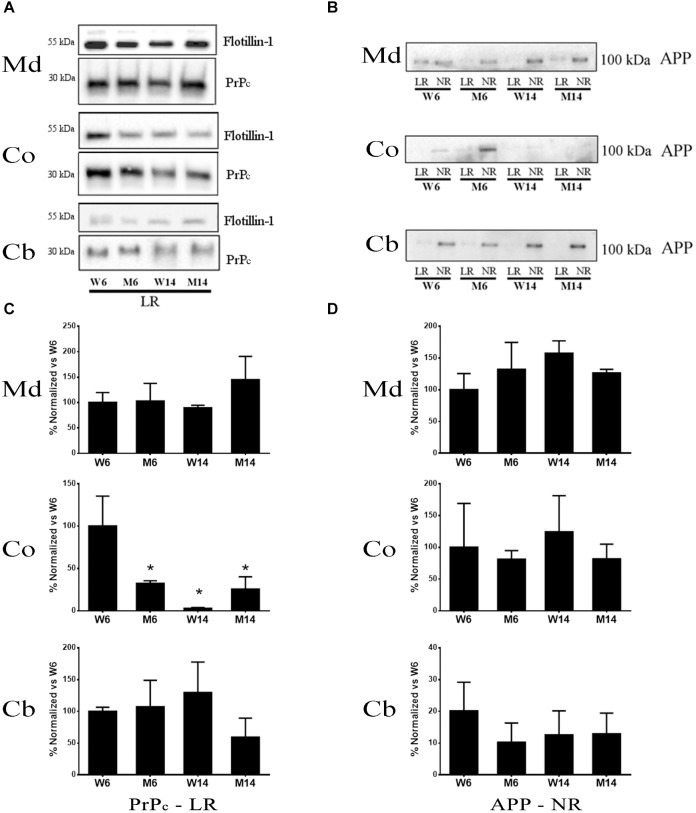
**(A)** Western blot analysis of the expression of PrP in lipid rafts (since this protein was absent or only slightly represented in non-raft fractions) from the four experimental cohorts. **(B)** Quantification of total PrP levels in lipid rafts normalized versus W6. (^∗^*p* < 0.05, One-way ANOVA with Newman–Keuls multiple comparison test vs. W6). **(C)** Western blot analysis of the expression of APP in lipid rafts and non-raft fractions from the four experimental cohorts. **(D)** Quantification of total APP levels in non-raft fractions normalized vs. W6 (*N* = 4).

PrPc is a GPI-anchored protein whose interaction with GM1 is crucial for the protein partitioning and conformation (Botto et al., 2014). The distinct compartmentalization of α-syn and the glutamatergic receptors NMDAR2B and GluR5 observed related to age and neurotoxic parameters led us to analyze PrPc distribution in the membrane fractions. We performed Western blot analyses to establish PrPc levels in our experimental cohorts (Figure 8A). In addition, we included APP in our analysis as a relevant pathological comparison of other neurodegenerative proteinopathies such as AD (Figure 8B) (Schengrund, 2010; Marin et al., 2016). Interestingly, PrPc showed a striking decrease in cortical LR fractions following both aging and pharmacological injury as compared to young healthy controls (Figure 8C). Moreover, we observed a similar pattern in the distribution of APP and PrPc in the Md LRs of aged and MPTP-treated mice (Figure 8C,D). In contrast, PrPc was absent of NR fractions.

### Distinct Partitioning and Aggregation of α-syn in Membrane Fractions of PD Brains

**FIGURE 9:**
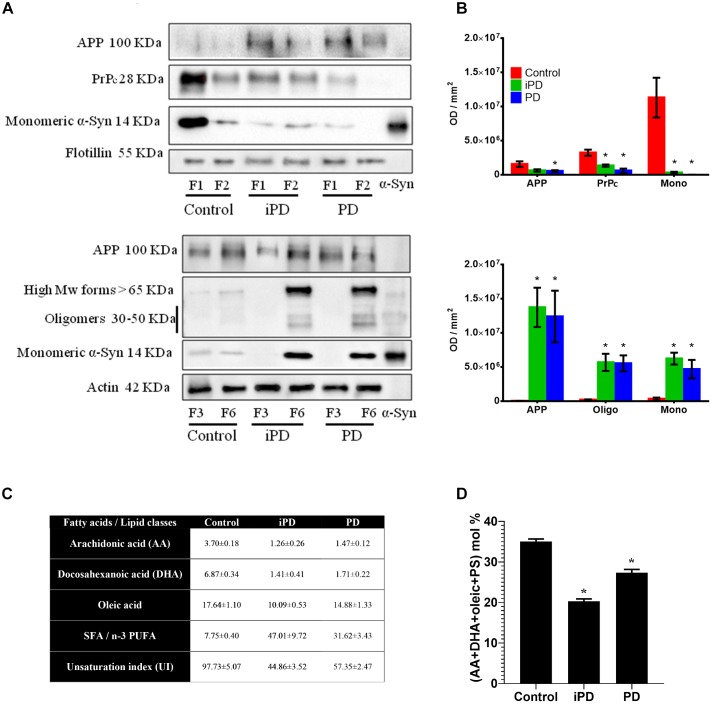
**(A)** Comparative Western blot analysis of protein markers in LR (F1 and F2) and NR (F3 and F6) fractions of human frontal cortex from three experimental cohorts: control, incidental Parkinson’s disease (iPD) and Parkinson’s disease (PD). Top: Samples were immunoblotted for the amyloid precursor protein (APP), the prion protein (PrP) and the monomeric form of α-syn in lipid rafts. Flotillin was used as control of LR content. Bottom: Samples were immunoblotted for APP, and the monomeric, oligomeric and high Mw forms of the α-syn in non-raft fractions. Actin was used as a control. **(B)** Top: Quantification of APP, PrPc and the monomeric form of α-syn in lipid rafts. Bottom: Quantification of APP, and the monomeric, oligomeric and high Mw forms of α-syn in NR fractions. Proteins were quantified and normalized against flotillin in F1 and F2 (top) and against actin in F3 and F6 (bottom). (^∗^*p* < 0.05, One-way ANOVA with Newman–Keuls multiple comparison test vs. W6). **(C)** Quantification of fatty acids and lipid classes from lipid rafts in control humans and patients suffering incidental Parkinson’s (iPD) and Parkinson’s disease (PD) (adapted from Fabelo et al., 2011). AA, DHA and oleic are expressed as average mole percentage ± statistical error, and ratios derived from them and other, not shown fatty acid species. **(D)** Cumulative mole percentage of arachidonic and docosahexanoic acids (AA and DHA, respectively), oleic acid and phosphatidylserine (PS) in lipid rafts from control humans and patients suffering iPD and PD. AA, DHA and oleic are expressed as average mole percentage ± statistical error. ^∗^*p* < 0.05, One-way ANOVA with Newman–Keuls multiple comparison test vs. control (*N* = 5).

We further evaluated by immunoblotting α-syn distribution and conformation in membrane fractions from the frontal cortex of incidental Parkinson’s disease (iPD) and PD brains. Age-matched healthy controls were used for comparison (Figure 9A,B). These samples had previously shown profound pathological changes in the lipid composition of LR (F1-F2) fractions and significant modifications in their physicochemical properties (Fabelo et al., 2011; Marin et al., 2017). Monomeric α-syn was shown highly abundant in LR from control samples. This protein was significantly observed to decrease in iPD and PD brains. On the contrary, monomeric α-syn was increased in NR fractions with the progression of the pathology (Figure 9A,B). α-Syn displacement to the non-raft fractions (F3-F6) remarkably correlated with an appearance of α-syn high Mw species (>65 kDa) of iPD and PD samples, as quantified by bar graphs normalized to control samples.

To further characterize the putative partitioning of other synuclein pathological protein biomarkers of the study, we examined the presence of PrPc and APP in these membrane compartments (Figure 9A,B). The immunoblotting results on the same experimental samples revealed a progressive reduction of PrPc content in F1-F2 that correlated with PD pathology. PrPc was undetected in NR pellets (data not shown). Moreover, APP showed a shifting to NR in both iPD and PD subjects as compared to healthy controls.

We have previously demonstrated a highly significant detriment in PUFA (AA + DHA) and oleic acid levels that correlated with an unbalanced UI (Fabelo et al., 2011). For further clarification, these values are succinctly summarized in Figure 9C. Based upon our data, we analyzed whether the progressive reduction in monomeric α-syn observed in cortical LR with the progression of PD correlated with the combinatorial variations in the levels of the main lipid factors reported to affect α-syn configuration at the neuronal membrane (Figure 9D). Interestingly, the accumulated variations of AA, DHA, oleic acid and PS accounted for ∼49% of total lipid changes in iPD as compared with age-matched controls. Although not conclusively demonstrated here, this lipid species rearrangement may be a factor contributing to α-syn insertion in these membrane microstructures.

**FIGURE 10:**
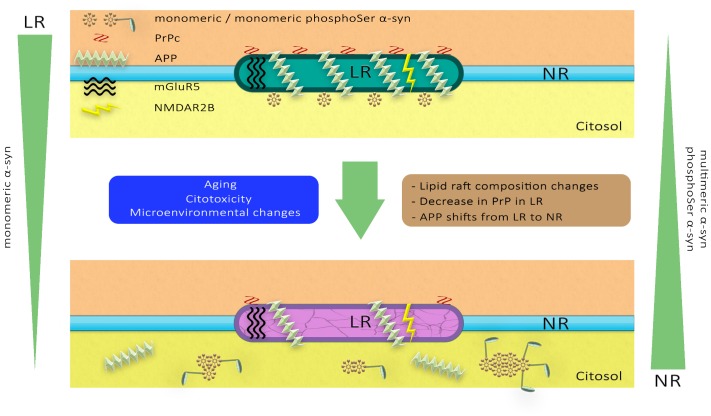
A hypothetical model of lipid raft alterations and their functional and structural consequences. Diverse stimuli may affect the strictly regulated chemical composition of the lipid raft microdomains, altering their physicochemical properties and facilitating pathological changes in proteins such as glutamatergic receptors, PrPc and APP. These molecular anomalies in neuronal lipid rafts may promote changes in the aggregation and phosphorylation of α-synuclein associated with PD pathology.

Overall, these observations indicate that the distribution of α-syn in membranous compartments differs during aging and following MPTP neurotoxicity. Furthermore, pathological impairment of lipid microenvironment may also affect membrane partitioning of other raft-associated proteins involved in distinct proteinopathies, such as glutamatergic receptors, PrPc and APP. A schematic representation of the dynamics of these membrane proteins in the PD pathological scenario has been illustrated in Figure 10.

## Discussion

Increasing evidence indicates that cell membrane microdomains with a specific, well-regulated lipid and protein composition such as LR may be essential for neuronal health. Even minor alterations in these structures can trigger pathological effects by modifying the way lipids and proteins interact, thereby affecting neurotoxic and neuroprotective responses. Indeed, our previous studies have demonstrated that instability of raft microdomains appears to be a crucial early event in the development of synucleinopathies (Fabelo et al., 2011; Marin et al., 2016, 2017). In this sense, a plausible hypothesis is that the membrane lipid microenvironment may determine the modulation and trafficking of distinct α-syn molecular configurations. To explore this issue, we have investigated here the putative molecular alterations of distinct membrane lipid fractions, LR and NR, and their subsequent impact on α-syn dynamics. Our findings demonstrate that rearrangement of membrane gangliosides, PUFA, Cho, SFA and phospholipids induced by brain aging and PD injury have profound effects on α-syn distribution and oligomerization in abnormal structures. Furthermore, these changes in protein trafficking may be extended to other biomarkers related to neurodegenerative diseases, such as PrPc, APP and glutamatergic receptors.

We used a well-established murine model of PD (Bezard et al., 1997; Jackson-Lewis and Przedborski, 2007; Meredith and Rademacher, 2011; Schlachetzki et al., 2016). This paradigm is based on the loss of dopaminergic neurons in SNpc and consequent motor deficits following MPTP neurotoxin administration (Schmidt and Ferger, 2001; Jackson-Lewis and Przedborski, 2007). In line with previous observations (Seniuk et al., 1990; Mitsumoto et al., 1998), we detected here a significant decline of TH-IR neurons in the SNpc of MPTP-treated mice. Similarly, a significant TH-IR loss was shown in SNpc of untreated 14-months aged mice as compared to 6-months aged ones. These observations reflect a potential progressive detriment of dopaminergic activity in this brain area caused by mere aging. This mouse phenotype is in agreement with previous studies of age-dependent changes in gene expression that have suggested the existence of an accelerated detriment of SNpc in aged mice (Gao et al., 2013), and a loss of TH-IR neurons and dopamine production in old animals (Credle et al., 2015).

In the present study, lipid analyses of membrane fractionation in midbrain (Md) and cortex (Co) from MPTP-treated animals reflected alterations in representative lipid classes of these brain regions, including impairment in distinct ganglioside species. Noticeably, gangliosides have been implicated as regulators of α-syn pathology (Ariga, 2014; Schnaar, 2016), and binding of this protein to GM1 inhibits fibrillation (Martinez et al., 2007). In this work, we did not observe a significant unbalance of GM1 present in raft fractions following MPTP treatment. However, we observed anomalies in the levels of other ganglioside species (GD1a, GD1b, and GT1b), in particular in aged mice exposed to MPTP. Although the influence of GD1a, GD1b, and GT1b on α-syn membrane regulation has been little explored (Badawy et al., 2018), these data indicate that, apart from GM1, the specific rearrangement of other ganglioside lipid classes may participate in the partitioning and conformational structure of α-syn. For instance, folding of this protein is enhanced by distinct gangliosides in a specific manner (Gaspar et al., 2018).

Together with ganglioside species, we detected here a disproportion of PUFA, Cho, SFAs and phospholipids ratio during aging that were accelerated by MPTP injury. Indeed, comparison of the lipid bundles collected from membranous compartments evidenced a conversed variation in the levels of these lipid classes induced by aging and neurotoxicity. For instance, the standard proportions of Cho/PUFA, PL/PUFA and Cho/PUFA showed downward trends in LR but significant upward variations in NR. Treated animals also displayed significant changes in total polar lipids (mainly phospholipids, PL) which varied steadily with age and phenotype, in particular in NR fractions. Moreover, SFA/PUFA ratio was highly unbalanced as compared to the healthy young cohort used as control. In parallel with these changes, the UI was particularly reduced in aged animals. This phenomenon may directly influence the ability of neuron membranes to buffer oxidative insult associated with neurotoxicity. Furthermore, UI detriment indicates the presence of a more viscous and liquid-ordered microstructures in MPTP animals (Krishna and Periasamy, 1999), a phenomenon that may affect protein configuration and dynamics of protein assemblies embedded in these lipid matrices, including α-syn. The fact that neurotoxic treatment affected membrane microdomain stability even in 6-month age cohort indicates that these structural changes may be at the basis of abnormal molecular aberrations. Nevertheless, membrane impairment under pathological conditions may reach a maximal physicochemical limit that may be counteracted by aging as the main component of neuronal dysfunction.

Previous data has revealed that polyunsaturated acyl groups, in particular AA and DHA, can induce multimerization of α-syn in phospholipid vesicles (Perrin et al., 2001). Taking into account the considerable variation of these PUFA in membrane compartments induced by MPTP treatment, we further characterized whether these molecular fluctuations may generate the basis for α-syn oligomerization. Binding affinity of α-syn to lipid microdomains requires the combination of PUFA (AA or DHA), oleic acid and PS (Kubo et al., 2005). In our work, levels of PS, and no other phospholipids, were shown to be profoundly affected by MPTP toxicity in both midbrain and cortex from NR extracts. More interestingly, as compared to other lipid subsets, the molecular combination of PS with AA, DHA and oleic acid accounted for ∼60% of the total NR lipid matrix associated with MPTP lesion. The requirement for both mono- and polyunsaturated acyl chains indicates that α-syn configuration may strongly depend on a particular biphasic transition to facilitate protein-lipid interactions. It is worth mentioning that, even if Cho is a main component for raft stability, this sterol does not appear to be a main factor for specific binding and modification of α-syn by raft lipid clustering (Kubo et al., 2005). Even though some reports have claimed that high Cho causes the accumulation of α-syn in PD (Abramov et al., 2011; Galvagnion et al., 2015), other studies have demonstrated that there is not a direct link between Cho levels and PD development (Paul et al., 2015; Doria et al., 2016). These apparent controversies may be explained by alteration in the local architecture of membrane microdomains caused by trafficking of distinct lipid species. In this order of ideas, Cho is preferentially located in the exofacial leaflet of the membrane and shows a poor affinity for DHA. It has been proposed that even subtle changes in PUFA content may have profound consequences in membrane order and fluidity, phase behavior and the dynamic partitioning of proteins embedded in this microenvironment (Stillwell and Wassall, 2003; Shaikh et al., 2004). These changes may indeed account for the specific association of α-syn with membranous compartments, thereby altering its configuration. At another level, α-syn may also in reverse influence the structural and functioning of lipid stability of membranes, thereby enhancing abnormal lipid rearrangement that may potentiate the neurotoxic effect (Zhu and Fink, 2003; Hellstrand et al., 2013). In this sense, α-syn knock-out mice exhibit increased levels of PUFA by still unknown mechanistic strategies (Sharon et al., 2003b).

Related to the cerebellum, some lipid changes were also observed, associated with alterations in both Cho and PUFA levels. However, as expected, most changes in this region were associated with aging rather than to neurotoxin exposure. Noticeably, induction of MPTP neurotoxicity requires the expression of dopamine transporter (DAT) to access intracellular compartments (Maries et al., 2003; Meredith and Rademacher, 2011). In this sense, cerebellar neurons are deprived of DAT in these mice and, therefore, may be resistant to MPTP injury (unpublished results). Furthermore, lipid matrix imbalance in membrane microstructures related to MPTP phenotype closely resembles those previously reported by our group in APP/PS1 transgenic mice, a model of AD degeneration. In these mice, Cho and PUFA disproportion, together with a lower unsaturation capability, were also reported with the progression of aging and AD pathology (Fabelo et al., 2012). More remarkable, profound changes in cortical microdomains have been previously observed by our group in human synucleopathies, such as PD and DLB. In LR from human cortex, DHA (22:6n-3), AA (20:4n-6) and oleic acid (18:1n-9) were shown to be significantly reduced even at the preamble of these diseases, in parallel with an increase in SFA (16:0 and 18:0) (Fabelo et al., 2011). Likewise, accelerated LR aging has been previously characterized in early stages of human AD brains, consistently observing similar changes in PUFA/SFA ratio with the progression of the pathology (Diaz et al., 2018). Overall, these data indicate that PD-like lesion accelerates the normal aging process of lipid changes in cognitive and motor brain areas, provoking a premature triggering of neuropathological membrane features.

Our present proteomic analyses of α-syn in experimental mice cohorts revealed the accumulation of high MW forms of this protein in the three nerve regions analyzed during aging and toxic treatment. α-syn multimers were particularly abundant in cortical NR compartments, although they were also visible in Md areas. Conversely, the amount of this protein was negligible in raft fractions, where only a faint representation of monomeric α-syn was detected in young healthy controls. The high Mw α-syn aggregates found here have also been previously detected in the α-syn E57K transgenic mice (Rockenstein et al., 2014). However, other alternative rearrangements of the protein may be expected considering the exceedingly complex nature of α-syn biochemistry (Mor et al., 2016). Additionally, α-syn monomers were also detected in NR fractions. These monomeric forms have been previously described in normal mammalian cells, and represent α-syn native, metastable physiological forms of the protein (Bartels et al., 2011; Dettmer et al., 2015). It is worth mentioning that other cognitive areas not investigated here, such as the hippocampus, may also be affected by MPTP treatment. Thus, recent data has demonstrated the correlation between the post-translational modification and accumulation of membrane-associated α-syn with impaired hippocampal plasticity (Schlachetzki et al., 2016).

Interestingly, α-synuclein oligomers have been found in exosomes which are membrane nanovesicles secreted by cells, in the central nervous system (Danzer et al., 2012). Together with recent biophysical findings that toxic α-syn oligomerization reduces the ability of this protein to interact with lipid membranes depending upon their chemical composition (Gallea et al., 2018), this would suggest a potential spreading mechanism for multimeric α-synuclein isoforms with pathological relevance. Furthermore, MPTP phenotype may also increase lipid peroxidation at the membrane (Moldovan and Moldovan, 2004) by inhibiting mitochondrial complex I (Nicklas et al., 1985; Chan et al., 1991) and inducing the generation of reactive oxygen species (Zang and Misra, 1993). These cellular events may ultimately affect the stability of the monomeric α-syn membrane binding, thus facilitating oligomerization and modifying protein homeostasis.

In contrast with mice membranes, our data showed a high abundance of monomeric α-syn in raft domains from the human frontal cortex. However, this protein was largely diminished in cortical areas of both iPD and PD brains as compared to age-matched healthy controls. Interestingly, concomitant with monomeric α-syn reduction, we detected the appearance of high Mw α-syn aggregates in a similar pattern to MPTP-treated mice. Multimerization of this protein occurred in parallel with its trafficking to NR fractions of the pathological subjects. To the best of our knowledge, this is the first demonstration of α-syn multimeric distribution related to raft membrane compartments. Interestingly, α-syn multimer pattern may be common in different synucleopathies, as aggregated forms of this protein were also detected in the frontal cortex of cases with dementia with Lewy bodies (DLB) (Marin et al., 2017). DLB is another synucleinopathy characterized by widespread α-syn deposition in different brain regions, including cortical areas (McKeith et al., 2004; Jellinger, 2018; Jellinger and Korczyn, 2018). Although still little explored, α-syn aggregation in DLB may also be a consequence of the loss of this protein bound to LRs due to lipid constraints (Marin et al., 2017). Overall, these data are an indicative of the compartmentalization of this protein in NRs related to synucleinopathies, correlating with the lipid impairment observed in membrane microdomains (Fabelo et al., 2011; Marin et al., 2017). In agreement with this, previous studies have demonstrated that selective lipid loading determines the initial phases of α-syn aggregation, specifically dimers and trimers, providing the core seed for further complex aggregation (Cole et al., 2002). This is particularly interesting when considering the trends observed in glutamatergic receptors after MPTP treatment in murine subjects. Previous studies have found that α-syn regulates both membrane NMDA levels and the internalization of NMDA receptors (Cheng et al., 2011; Chen et al., 2015). Furthermore, the rotenone toxin, which impairs mitochondrial function in a way analog to MPTP treatment, has been reported to induce a decrease in NMDA2B levels (Navarria et al., 2015). Altogether, these findings suggest a role of the aforementioned selective lipid loading in the regulation of glutamatergic membrane receptors via the preservation of monomeric α-syn. This molecular mechanism may be potentially take part in the PD pathogenesis when lipid content is altered thus enhanced protein multimerization.

Another consequence of altered structure of lipid microstructures during PD neuropathology is that it may provide the molecular environment for further toxic α-syn phosphorylation. For instance, phosphorylation in S129 residue (pS129) has been found highly represented in α-syn aggregates of various synucleinopathies (Fujiwara et al., 2002). In this work, 2-D immunoblotting assays to detect pS129 α-syn demonstrated that this phosphorylated form was mainly present in NRs of aged mice. This fact was reflected by the increase in the number of recurring high Mw isoforms with distinctive p*I* (6.7–8.4) observed by immunoblotting. To our knowledge, this represents the first illustration of α-syn pattern of post-transductional modification in membrane fractions. A similar phosphorylation trend was also observed in young mice exposed to PD-like neurotoxicity. Moreover, as expected, a significant reduction of p*I* 5.0 isoform corresponding to the unmodified α-syn monomer (Gould et al., 2014; Luth et al., 2015) was detected in aged brains. Thus, α-syn presence in NR membrane fractions may involve pS129 α-syn enhancement in correlation with α-syn aggregation production. These results reflect that α-syn phosphorylation in this residue is age-dependent, according to local microenvironmental changes. Thus, pS129 α-syn appears to correlate with lipid alterations occurring following MPTP injury, further evidencing the coordination of α-syn processing with neuronal membrane lipid homeostasis (Samuel et al., 2016). Moreover, although not explored in this work, other post-translational modifications of α-syn, including phosphorylation at Ser87 and Tyr 125 may also be involved in α-syn toxic assemblies (Xu et al., 2015).

Mounting evidence suggests that the key self-aggregating proteins in different proteinopathies, such as Aβ, PrPc and α-syn may share similar biophysical properties that affect the biochemical interrelations with membrane-integrated molecular compounds (Goedert, 2015; Ugalde et al., 2016; Goedert et al., 2017). In this sense, LR platforms are considered key sites in the modulation of amyloid-like seeding of several neurodegenerative diseases, including PD (Kazlauskaite et al., 2003; Arbor et al., 2016). In the present study, in parallel with the shifting of monomeric α-syn from LR to NR microstructures, we also observed the decrease of PrPc with the progression of PD in both mice and human samples. Aging process also modified the compartmentalization of PrPc. This is in correlation with previous data where a decrease of PrPc was reported during human aging and AD-related neurodegeneration (Whitehouse et al., 2010). However, PrPc trafficking behavior may be region-dependent, as other data have reported a slight increase of this protein in the hippocampus of aged mice (Agostini et al., 2013). These apparent discrepancies may be explained by functional differences between brain regions.

An interesting recent finding is that α-syn and PrPc markers appear to be interacting in the neuronal membrane (Ferreira et al., 2017). In our experiments, we did not observe a significant interaction of these two markers in raft samples, most probably due to the small amount of protein content represented in the membrane isolates (data not shown). The fact that these two protein markers follow a similar detriment in mice and human LRs suggests that lipid disturbances correlating with aging and PD neurotoxicity may affect interactions between these proteins. We postulate that a key lipid species in the pathological behavior of both PrPc and α-syn at the cell surface may be GM1. PrPc binding to this ganglioside is a prerequisite in the mechanisms predisposing to neuropathology (Botto et al., 2014). GM1 also promotes toxic α-syn oligomerization (Martinez et al., 2007) although other ganglioside species may also be involved (Badawy et al., 2018). Furthermore, clustering of amyloidogenic proteins may enhance toxic cross-seeding. Thus, scrapie PrP and Aβ have been shown to seed the formation of α-syn cytosolic aggregates in transgenic mouse models (Morales et al., 2010; Mougenot et al., 2011), a phenomenon that may drive disease progression. Also, α-syn and tau interactions at the cellular level appear to contribute to both tauopathies and synucleinopathies, indicating a synergistic effect between these two protein associations in the progression of the pathological features of these diseases (Esposito et al., 2007; Lassen et al., 2016; Li et al., 2016).

Moreover, other proteins clustering in raft multimeric associations may be affected by changes in membrane microstructures related to cognitive and motor impairment. For instance, α-syn/PrPc interaction is required to induce synaptic impairment through the activation of glutamatergic receptors (Um et al., 2012; Ferreira et al., 2017). We analyzed the distribution of APP and glutamatergic receptors in different LR and NR samples in this study. Interestingly, we detected a reduction of both NMDAR2B and mGluR5 correlating with aging and PD-like in mice. In addition, APP followed a similar shifting to NR fractions in PD brains. This protein redistribution may be a consequence of raft architecture rearrangements that may favor the amyloidogenic cleavage of APP (Fabelo et al., 2014). Overall, these data support the complexity of α-syn interactions to multiple partners within plasma membrane compartments, a fact that may affect disease-related events.

Overall, our data provide a further insight into the microenvironmental changes occurring during age and synucleinopathy toxicity taking place in distinct plasma membrane compartments. Alterations in the lipid matrix of raft membrane compartments may enhance α-syn trafficking, concomitantly with an enhancement of toxic conformational rearrangement and increased phosphorylation. The aberrant lipid modifications in raft architecture may promote the rearrangement of other pathologically relevant protein species, such as PrPc, glutamatergic receptors and APP that may ultimately contribute to neuronal impairment. In an attempt to illustrate this potential molecular scenario, we have depicted a schematic representation of the different markers analyzed here (Figure 10).

Further studies will be required to investigate the implication of self-aggregated proteins such as α-syn and other PD-linked proteins in neuronal LR, and the manner these membrane microstructures may be involved in both neuronal preservation and the pathogenesis of PD.

## Ethics Statement

This study was carried out in accordance with the recommendations and guidelines set by the European Union (86/609/UE) and in accordance with the current legal regulation of Spain Ley 32/2007, RD 53/2013, Ley 9/2003 y RD 178/2004). The protocol was approved by the Ethics Committee of La Laguna University and the Consejería de Agricultura, Ganadería y Pesca (Canarian government). This study was carried out in accordance with the recommendations of the ethical principles for medical research involving humans (Helsinki declaration) and subsequent amendments (Edinburgh, 2000), and the law 14/2007 for biomedical research, with written informed consent from all subjects. All subjects gave written informed consent in accordance with the Declaration of Helsinki. The protocol was approved by the Ethics Committee of the University of Barcelona (Spain).

## Author Contributions

RM has the main leader of the research. AC-A performed 60% of the experimental work. DP made all the statistical analyses and wrote some parts of the manuscript. DR-B performed the lipid analyses. VC-S made the extraction of brain tissue in animal models. PG-E and IF were in charge of handling human brain samples and lipid raft extractions. RP-A performed the protein analyses of human samples. MH provided the anti-ganglioside antibodies. MD was the responsible and mentor of lipid isolation and mice experimental models.

## Conflict of Interest Statement

The authors declare that the research was conducted in the absence of any commercial or financial relationships that could be construed as a potential conflict of interest.

## Supplementary Material

FIGURE S1 Slot-blot analysis of ganglioside GM1 in lipid raft (LR) and non-raft (NR) fractions. Three different CNS functional areas were studied in the four experimental cohorts: midbrain (Md), cortex (Co) and cerebellum (Cb). Figure shows all four animals used for quantification. Graphs showing the quantification are included in Figure 2.

FIGURE S2 Quantification of fatty acids and lipid classes of midbrain (Md) cortex (Co) and cerebellum (Cb) across the experimental cohorts in lipid raft (LR) and non-raft (NR) fractions. Cholesterol (CHO), arachidonic acid (20:4n-6) and docosahexanoic acid (22:3n-6) (AA and DHA, respectively), oleic acid (18:1n-9), phosphatidylserine (PS), total phospholipids (PL), saturated, polyunsaturated and unsaturated fatty acids (SFA, PUFA and UFA, respectively), and sulfatide are expressed as average mole percentage ± statistical error, and ratios derived from them and other, not shown, fatty acid species (*N* = 4).

FIGURE S3 **(A)** Lipid profiles of midbrain (Md) cortex (Co) and cerebellum (Cb) across the experimental cohorts in lipid raft (LR) fractions, following aging (M6 vs. W6, left) and MPTP treatment (M6 vs. W6 and M14 vs. W14, right). Average mole percentage data from M6 and M14 were normalized to its controls (W6 and W14, respectively) and plotted as such. **(B)** Lipid profiles of midbrain (Md) cortex (Co) and cerebellum (Cb) across the experimental cohorts in non-raft (NR) fractions in aged (M6 vs. W6, left) and treated (M6 vs. W6 and M14 vs. W14, right) cohorts (*N* = 4). Average mole percentage data from M6 and M14 were normalized to its controls (W6 and W14, respectively) and plotted as such.

FIGURE S4 Bivariate relationship between the polyunsaturated acids (PUFA) AA and DHA, and phosphatidylserine (PS) in midbrain (Md) cortex (Co) and cerebellum (Cb) across the experimental cohorts in non-raft (NR) vs. lipid raft (LR) fractions. Values were calculated by the quotient of the product of the square root of the mole percentage of DHA + AA by the mole percentage of PS in NR between LR. ^∗^*p* < 0.05, One-way ANOVA with Newman–Keuls multiple comparison test vs. W6 (*N* = 4).

## Abbreviations

α-syn: alphα-synuclein
AA: 20:4, arachidonic acid
Cho: cholesterol
DHA: 22:6, docosahexaenoic acid
LRs: lipid rafts
MPTP: 1-Methyl-4-phenyl 1,2,3,6-tetrahydropyridine
PS: phosphatidylserine
PUFA: polyunsaturated fatty acids
SFA: saturated fatty acids
TH: tyrosine hydroxylase
