# Supplementary figures and images for: Differential Aggregation and Phosphorylation of Alpha Synuclein in Membrane Compartments Associated With Parkinson Disease
Source: Front Neurosci. 2019 Apr 24;13:382. doi: 10.3389/fnins.2019.00382 (PMC6491821; doi:10.3389/fnins.2019.00382)

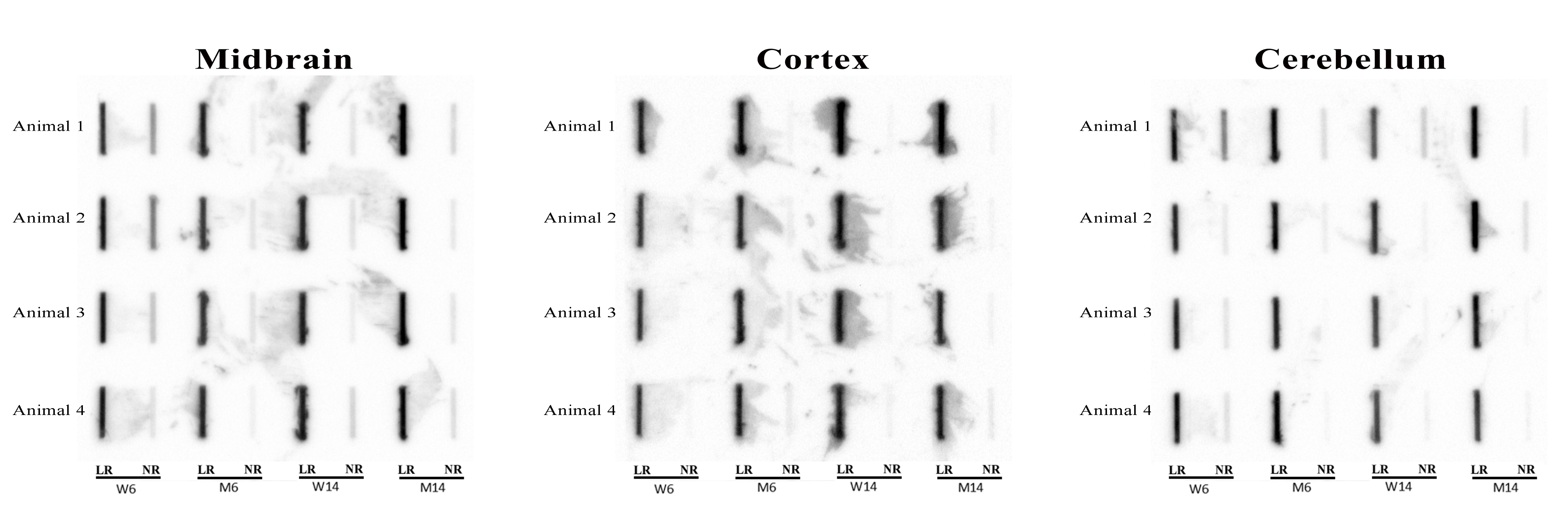

Supplement: FIGURE S1 — Slot-blot analysis of ganglioside GM1 in lipid raft (LR) and non-raft (NR) fractions. Three different CNS functional areas were studied in the four experimental cohorts: midbrain (Md), cortex (Co) and cerebellum (Cb). Figure shows all four animals used for quantification. Graphs showing the quantification are included in Figure 2. [file Image_1.JPEG]

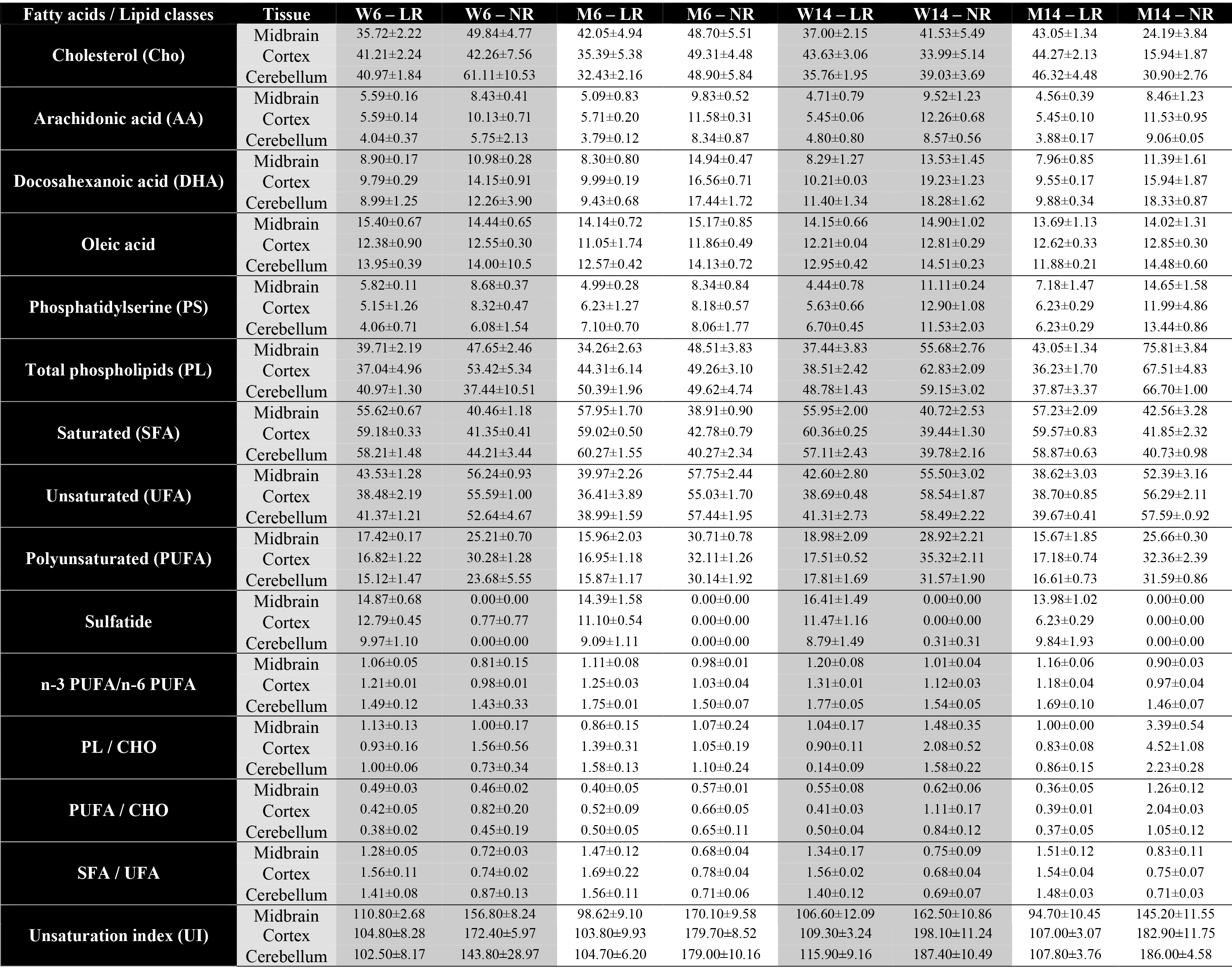

Supplement: FIGURE S2 — Quantification of fatty acids and lipid classes of midbrain (Md) cortex (Co) and cerebellum (Cb) across the experimental cohorts in lipid raft (LR) and non-raft (NR) fractions. Cholesterol (CHO), arachidonic acid (20:4n-6) and docosahexanoic acid (22:3n-6) (AA and DHA, respectively), oleic acid (18:1n-9), phosphatidylserine (PS), total phospholipids (PL), saturated, polyunsaturated and unsaturated fatty acids (SFA, PUFA and UFA, respectively), and sulfatide are expressed as average mole percentage ± statistical error, and ratios derived from them and other, not shown, fatty acid species (N = 4). [file Image_2.JPEG]

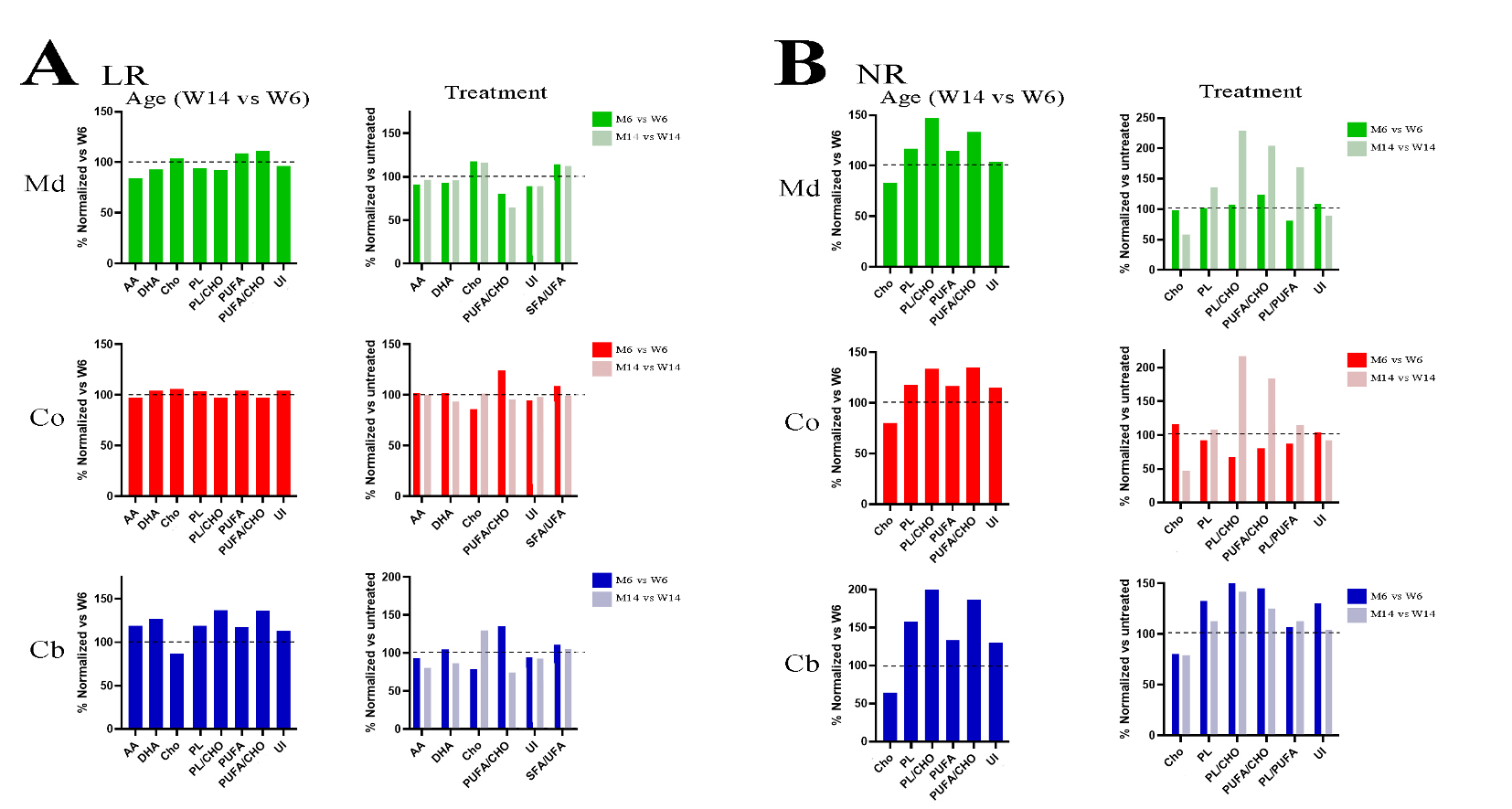

Supplement: FIGURE S3 — (A) Lipid profiles of midbrain (Md) cortex (Co) and cerebellum (Cb) across the experimental cohorts in lipid raft (LR) fractions, following aging (M6 vs. W6, left) and MPTP treatment (M6 vs. W6 and M14 vs. W14, right). Average mole percentage data from M6 and M14 were normalized to its controls (W6 and W14, respectively) and plotted as such. (B) Lipid profiles of midbrain (Md) cortex (Co) and cerebellum (Cb) across the experimental cohorts in non-raft (NR) fractions in aged (M6 vs. W6, left) and treated (M6 vs. W6 and M14 vs. W14, right) cohorts (N = 4). Average mole percentage data from M6 and M14 were normalized to its controls (W6 and W14, respectively) and plotted as such. [file Image_3.JPEG]

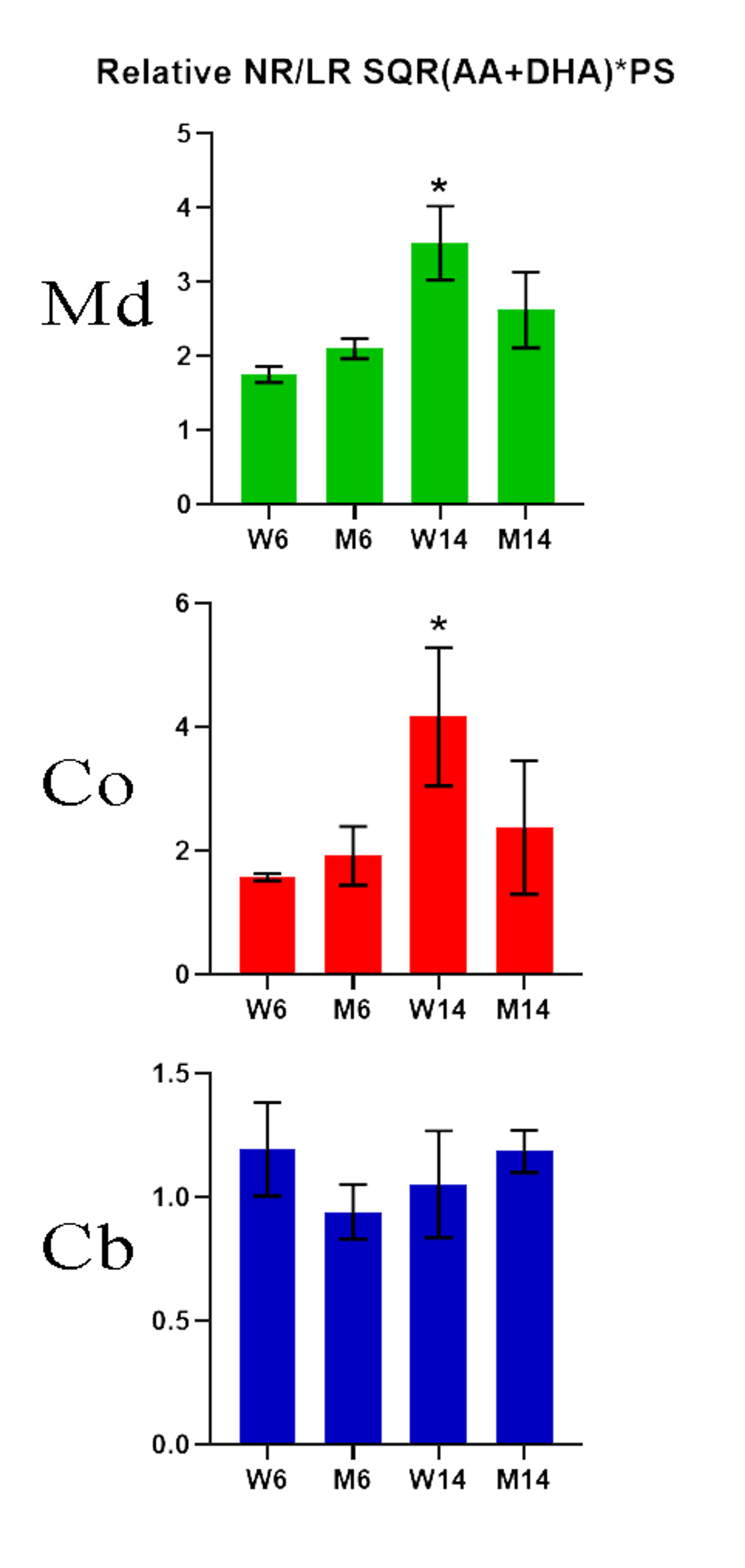

Supplement: FIGURE S4 — Bivariate relationship between the polyunsaturated acids (PUFA) AA and DHA, and phosphatidylserine (PS) in midbrain (Md) cortex (Co) and cerebellum (Cb) across the experimental cohorts in non-raft (NR) vs. lipid raft (LR) fractions. Values were calculated by the quotient of the product of the square root of the mole percentage of DHA + AA by the mole percentage of PS in NR between LR. ∗p < 0.05, One-way ANOVA with Newman–Keuls multiple comparison test vs. W6 (N = 4). [file Image_4.JPEG]
